# *In Vitro* Spermatogenesis in Explanted Adult Mouse Testis Tissues

**DOI:** 10.1371/journal.pone.0130171

**Published:** 2015-06-12

**Authors:** Takuya Sato, Kumiko Katagiri, Kazuaki Kojima, Mitsuru Komeya, Masahiro Yao, Takehiko Ogawa

**Affiliations:** 1 Laboratory of Proteomics, Institute of Molecular Medicine and Life Science, Yokohama City University Association of Medical Science, Yokohama, Japan; 2 Department of Urology, Yokohama City University Graduate School of Medicine, Yokohama, Japan; University Hospital of Münster, GERMANY

## Abstract

Research on *in vitro* spermatogenesis is important for elucidating the spermatogenic mechanism. We previously developed an organ culture method which can support spermatogenesis from spermatogonial stem cells up to sperm formation using immature mouse testis tissues. In this study, we examined whether it is also applicable to mature testis tissues of adult mice. We used two lines of transgenic mice, *Acrosin-*GFP and *Gsg2*-GFP, which carry the marker GFP gene specific for meiotic and haploid cells, respectively. Testis tissue fragments of adult GFP mice, aged from 4 to 29 weeks old, which express GFP at full extension, were cultured in medium supplemented with 10% KSR or AlbuMAX. GFP expression decreased rapidly and became the lowest at 7 to 14 days of culture, but then slightly increased during the following culture period. This increase reflected *de novo* spermatogenesis, confirmed by BrdU labeling in spermatocytes and spermatids. We also used vitamin A-deficient mice, whose testes contain only spermatogonia. The testes of those mice at 13-21 weeks old, showing no GFP expression at explantation, gained GFP expression during culturing, and spermatogenesis was confirmed histologically. In addition, the adult testis tissues of *Sl/Sl^d^* mutant mice, which lack spermatogenesis due to Kit ligand mutation, were cultured with recombinant Kit ligand to induce spermatogenesis up to haploid formation. Although the efficiency of spermatogenesis was lower than that of pup, present results showed that the organ culture method is effective for the culturing of mature adult mouse testis tissue, demonstrated by the induction of spermatogenesis from spermatogonia to haploid cells.

## Introduction

Spermatogenesis is a complex cellular process involving the proliferation and differentiation of male germ cells, which uniquely includes meiosis and spermiogenesis. The entire process takes 35 and 76 days in mice and humans, respectively, and needs a specific microenvironment produced by testicular somatic cells, Sertoli cells in particular [1, 2]. Thus, attempts to replicate it have long been unsuccessful under *in vitro* conditions. Considering that the histological architecture of the testis must be critical for the effective progression of mammalian spermatogenesis, we reasoned that organ culture methods, rather than cell culture methods, may prove more successful for developing a system for *in vitro* spermatogenesis [3]. During our testis tissue organ culture experiments, we found that supplements regularly used as serum replacement, Knockout Serum Replacement (KSR) and AlbuMAX, were effective to induce and promote spermatogenesis in tissue fragments of neonatal or pup mouse testis [4, 5]. In addition, we found that a particular type of spermatogenic failure was treatable by culturing testis tissues. We used a mutant mouse, *Sl/Sl* ^*d*^, as a model of such infertility because its cause is known at a molecular level: the absence of Kit ligand (KitL) on the surface of Sertoli cells, which abolishes spermatogenesis and leaves the testis with only primitive spermatogonia as germ cells. These testis tissues, when cultured with a sufficient amount of KitL in the culture medium, developed spermatogenesis, up to even producing fertility-proven haploid cells [6]. These recent findings are encouraging and could serve as a link to preclinical studies such as testis tissue culturing of primate models, which would be a basis for clinical applications in the future. There are, however, several critical challenges which remain to be overcome in order to make the organ culture method useful in clinical application for male infertility. As previous studies used immature mouse testis tissues exclusively, it has not been addressed if the culture method also works with mature adult testis tissues. Organ culture experiments to date have demonstrated that culturing adult tissues has been a challenge for most tissues and organs [7–10]. In this study, we tested whether our organ culture method is also applicable to mature testis tissues. We found that spermatogenesis can be induced in adult testis tissues from undifferentiated spermatogonia up to sperm formation, although its efficiency was far lower than that in neonatal tissues.

## Materials and Methods

### Mice and treatments

*Gsg2*-GFP transgenic mice [11], *Acr*-GFP transgenic mice [12, 13] (genetic background: ICR, C57BL/6 and their mixture), and WBB6F1-*Sl/Sl* ^*d*^ mice (Japan SLC) were used as testis tissue sources. Mice were housed in air-conditioned rooms, 24 ± 1°C and 55 ± 5%, with 14-hour light and 10-hour dark cycle. The MF hard pellets (Oriental Yeast Co., Ltd. Tokyo, Japan) were fed *ad libitum*. Drinking water was acidified to pH 2.8–3.0 by HCl. All animal experiments conformed to the Guide for the Care and Use of Laboratory Animals and were approved by the Institutional Committee of Laboratory Animal Experimentation (Animal Research Center of Yokohama City University, Yokohama, Japan).

The testis was decapsulated and divided by forceps into fragments of about 1–3 mm^3^. They were stored in culture medium on ice before use. In order to prepare VAD male mice, *Gsg2*-GFP or *Acr*-GFP females were fed a VAD diet (AIN-93G-based, Japan CLEA) for at least 4 weeks, and then mated with a non-treated male of the same line. The VAD diet was maintained throughout pregnancy until weaning. The male offspring continued to be fed the VAD diet until use [14]. Lack of GFP expression in the testis was confirmed before the initiation of the culture experiment. All animal experiments conformed to the Guide for Care and Use of Laboratory Animals and were approved by the Institutional Committee of Laboratory Animal Experimentation (Research Institute for Yokohama City University, Yokohama, Japan).

### Culture method

Agarose powder was dissolved in a DDW to 1.5% (w/v) and autoclaved. During the cooling, agarose solution was poured into 10-cm dishes to make gel. The gel was cut to about 1 cm square with about a 5-mm hight and this was used as a stand for testis tissue placement. The gels were submerged in the culture medium, αMEM with KSR (10%; Invitrogen, Carlsbad, CA, USA) or AlbuMAX (40 mg/mL; Invitrogen), for more than 6 hours before use. Tissue fragments were transferred to the surface of agarose gel that was half-soaked in the medium. Each gel was loaded with 1–3 tissue fragments. Medium change was performed once a week. The culture incubator was supplied with 5% carbon dioxide in air and maintained at 34°C. The protocol was reported previously in detail [15].

For *Sl/Sl* ^*d*^ mouse testis tissues, mouse stem cell factor (Kit ligand) (R&D Systems), and human colony stimulating factor-1 (CSF-1) (R&D Systems) were added to the medium at concentrations indicated in the text. For the BrdU incorporation assay, agarose gels were submerged in the culture medium supplemented with 3–10 μM BrdU.

### Gross and histological examination

Cultured tissues were periodically observed under a stereomicroscope (SZX12; Olympus, Tokyo, Japan or M205 FA; Leica, Wetzlar, Germany) to evaluate the presence of GFP-expressing cells, namely spermatocytes or spermatids. In order to semi-quantitativley measure the extent of GFP expression, we adopted a GFP-grading scale [4]. As the central area of cultured tissue usually does not express GFP, probably due the paucity of oxygen, we omitted that area from the evaluation. Then, the GFP expression observed in the peripheral area was classified into 6 grades. Grade 0 indicates no GFP expression. When a GFP-positive area covers 1 ~ 10% of the region, it is assigned Grade 1. The minimum GFP expression assigned as Grade 1 was at least a single stretch of GFP positive seminiferous tubule more than 1 mm long. As the GFP region increases to 11~30%, 31~50%, 51~70%, 71~90%, and 91~100%, the grade rises to 2, 3, 4, 5, and 6, respectively.

For histological examination, the specimens were fixed with Bouin’s fixative and embedded in paraffin. One section showing the largest cut surface was made for each specimen and stained with hematoxylin and eosin or Periodic acid-Schiff. Spermatogenesis-positive tubules were evaluated by calculating the percentage of seminiferous tubules containing differentiated (meiotic or more advanced) germ cells in these tissues. To search for spermatids and sperm, cultured tissues were mechanically dissociated using needles to release cells into the phosphate-buffered saline (PBS). The cell suspension was stained with Hoechst 33342 dye and observed with a microscope under GFP excitation light.

### Immunohistochemistry

Tissues were fixed with 4% paraformaldehyde in PBS at 4°C overnight. They were cryo-embedded in Tissue-Tek Optimal Cutting Temperature compound (Sakura Finetech, Tokyo, Japan) and cut into 7-μm-thick sections. The cryosections were washed with 0.2% PBT (0.2% Triton X-100 in PBS) four times and then treated with Image-iT FX Signal Enhancer (Invitrogen) for 30 min. Incubation with primary antibodies in 5% bovine serum albumin in 0.2% PBT was performed overnight at 4°C, followed by rinsing four times with 0.2% PBT, and then secondary antibodies were applied for 1 hour at room temperature. Nuclei were counterstained with Hoechst 33342 dye. Alexa 568-conjugated peanut agglutinin (PNA) was used to detect acrosomes (1:400, Invitrogen). Then, the immunologically stained samples were washed with 0.2% PBT four times and then mounted on slides in ProLong Gold (Invtirogen). The following were used as primary antibodies: rat anti-GFP antibody (1:1,000, Nacalai Tesque, Inc., Kyoto, Japan), mouse anti-BrdU antibody (Santa Cruz Biotechnology, Santa Cruz, CA, USA), and mouse anti-mouse sperm protein SP56 antibody (1:100; QED Bioscience, San Diego, CA, USA). The secondary antibodies used were: goat anti-mouse IgG, goat anti-rabbit IgG, and goat anti-rat IgG, conjugated with Alexa 488 or Alexa 555 (1:200; Invitrogen). For BrdU detection, cryosections were treated with 2 N HCl at 37°C for 30 min and washed with PBS before primary antibody treatment. Specimens were observed with a confocal laser microscope (FV-1000D; Olympus).

## Results

### Culturing of adult mouse testis tissues

**Fig 1:**
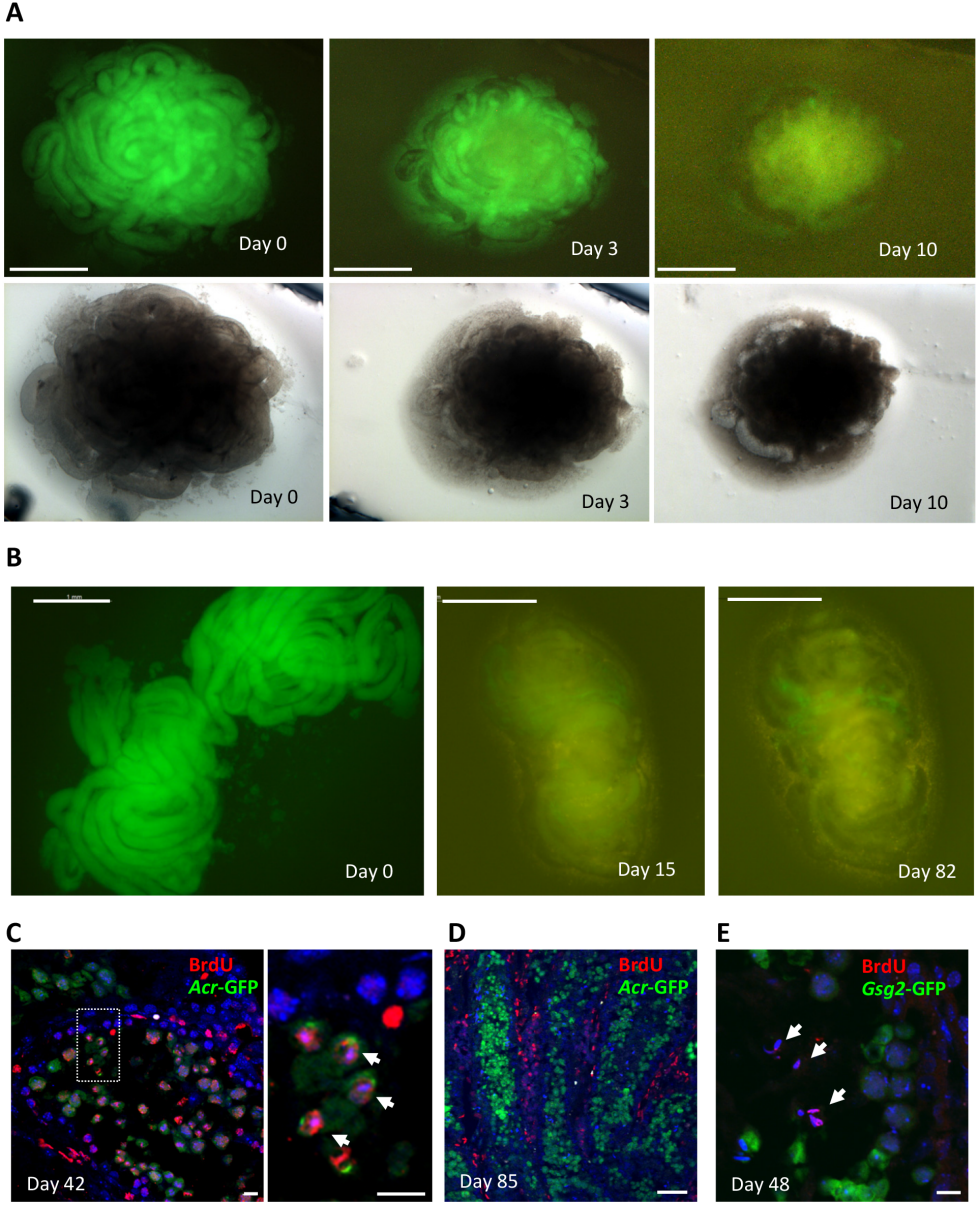
Stereomicroscopic view of cultured adult mouse testis tissues. (A) Six-week-old *Acr*-GFP mouse testis tissue fragments on culture days 0, 3, and 10. Upper row is GFP excitation view and lower row is bright view. (B) Six-week-old *Acr*-GFP mouse testis tissue fragments on culture days 0, 15, and 82. Weak GFP expression was maintained. (C-E) Immunohistochemistry of cultured tissues with antibodies to BrdU and GFP, counterstained with Hoechst. Testis tissue fragments of 6-week-old *Acr*-GFP (C and D) and 24-week-old *Gsg2*-GFP (E) mice were cultured, and then analyzed on days 42 (**C**), 85 (**D**), and 48 (**E**), respectively. The dashed rectangular area in the left picture of C is enlarged on the right. Arrows indicate BrdU-positive round spermatids in C and elongating spermatids in E. Scale bars, 1 mm (A, B); 10 μm (C, E); 50 μm (D).

Ten mice, 5 each of *Gsg2*-GFP and *Acr*-GFP transgenic, whose ages ranged from 4 to 28 weeks old, were used in the initial study (Table 1). Each testis tissue maximally expressed GFP at the start of culturing because the seminiferous tubules contained all stages of spermatogenic cells, including GFP-expressing spermatocytes and spermatids. We cut these testes into small pieces, 1–3 mm^3^, and cultured them with a gas-liquid interphase method. Soon after culture initiation, GFP expression reduced rapidly, mostly because the spermatogenic cells leaked out from the cut end of the seminiferous tubules in one or two days. This GFP reduction continued in the initial week and had almost disappeared or only slightly remained in the following week (Fig 1A). The structure of the seminiferous tubules was also deformed and seemed to have shrunk in general due to the leakage-related reduction of germ cells (Fig 1A). Nonetheless, a remnant of GFP, appearing as short strips or small dots along the seminiferous tubules, remained in certain cases for a long period, such as 82 days in our observation (Fig 1B). In fact, 25 tissue samples out of 74, using 10 mice, harbored such persisting GFP expression for more than 5 to 14 weeks (Table 1).

**Table 1:** Summary of adult mouse experiment.

Exp. ID	Age of mouse (weeks old)	GFP promoter	Medium supplement	Culture duration (weeks)	# Tissue fragment cultured	# Tissue with persisting GFP expression	# Tissue lost GFP expression
1	5	Gsg2	KSR	10	4	2	2
2	4	Gsg2	KSR	7	6	4	2
3	4	Acr	KSR	11	4	3	1
4	4	Gsg2	KSR	7	12	8	4
5	5	Gsg2	KSR	10	5	2	3
6	5	Acr	KSR	14	8	2	6
7	28	Gsg2	KSR	7	2	1	1
8	7	Acr	KSR	6	15	1	14
9	6	Acr	KSR	8	5	2	3
10	12	Acr	KSR	8	13	0	13
					74	25	49

In order to clarify whether such GFP expression reflected *de novo* spermatogenesis or mere remnants of GFP protein which may have been present since the initial phase of cultivation, we used 5-bromo-2'-deoxyuridine (BrdU) to label spermatogonia and identify their daughter cells during the subsequent culture period. Two sets of experiment, using 6- and 27-week-old mice, were undertaken. In the first experiment using a 6-week-old mouse, 13 testis tissue fragments were cultured. BrdU was added to the medium at the start of culturing, which was changed in 24 hours to a new BrdU-free medium. Two tissue fragments were removed for immunohistology on culture day 42. There were many BrdU-positive germ cells, including round spermatids (Fig 1C). In the remaining samples which were left for a longer culture period and processed for immunohistology on culture day 85, BrdU was found to be positive on interstitial cells but not germ cells in the seminiferous tubules, indicating that BrdU-positive germ cells went through spermatogenesis and then disappeared (Fig 1D). In the second experiment using a 28-week-old mouse, 2 testis tissues were cultured. BrdU was added to the culture medium during culture days 14 to 16, for 48 hours. On day 48, 1 of the 2 tissues which slightly expressed GFP was processed for immunohistology, showing many round and elongating spermatids. Among those haploid cells, only elongating spermatids stained positive for BrdU, indicating that these elongating spermatids came from spermatogonia that existed on days 14 to 16 (Fig 1E). These results demonstrated that spermatogonia and/or pre-leptotene spermatocytes, which are germ cells incorporating BrdU, differentiated into spermatids in the cultured adult testis tissues.

### Vitamin A-deficient mouse experiments

**Fig 2:**
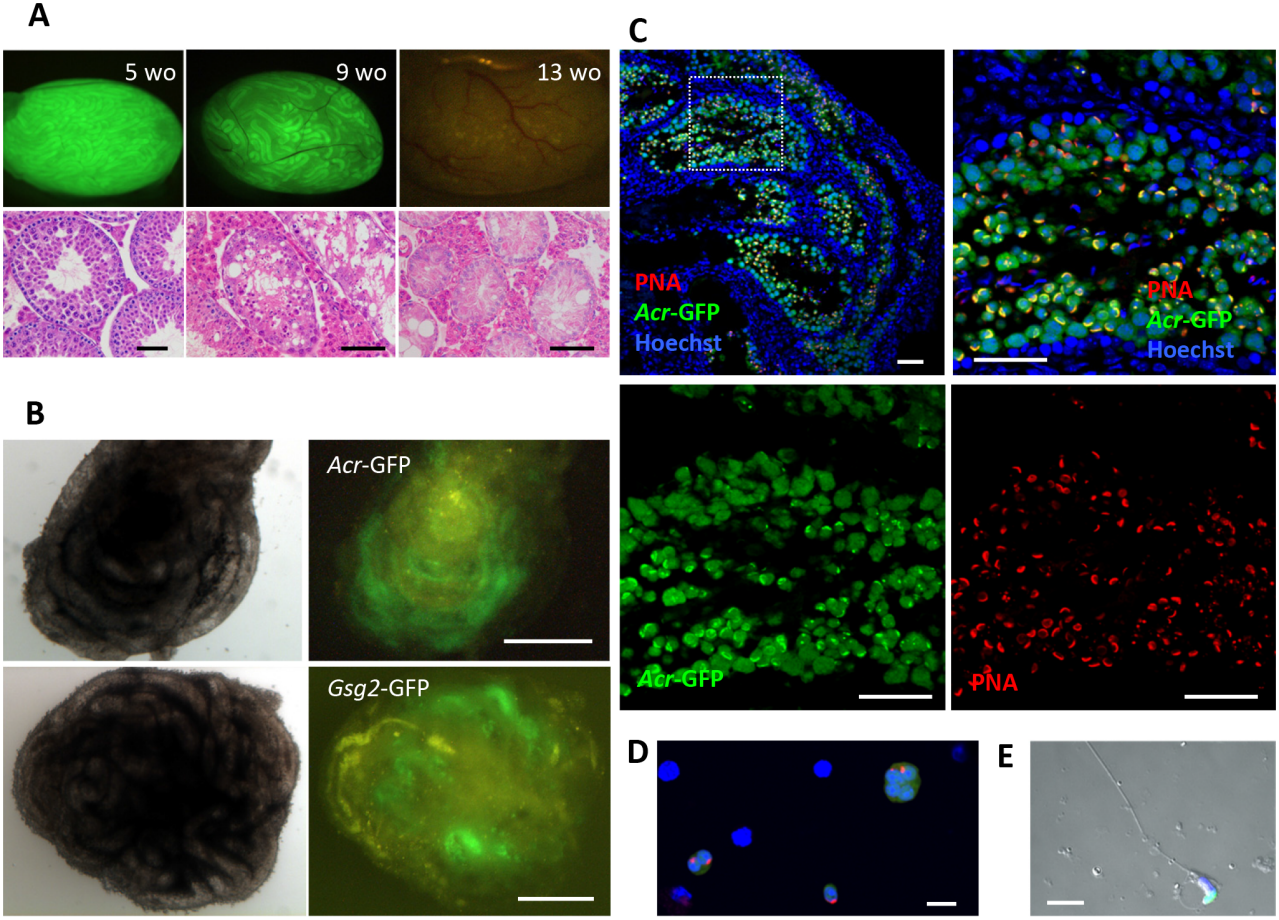
VAD experiment. (A) Testes of VAD treated *Gsg2*-GFP mice at, 5, 9 and 13 weeks old, showing decreasing GFP expression. Histology shows a corresponding extent of spermatogenesis on HE staining. At 13 weeks old, the GFP had completely disappeared and differentiating germ cells were totally removed. (B) Testis tissues of a VAD-treated *Acr*-GFP mouse, 24 weeks old, upper two images, and a VAD-treated *Gsg2*-GFP mouse, 13 weeks old, lower two images, regained GFP expression during culturing. Images were obtained on culture days 23 (upper two) and 70 (left two), respectively. (C) Immunohistochemistry of cultured testis tissue, on culture day 45, derived from a VAD-treated *Acr*-GFP 23-week-old mouse. Antibodies used were against GFP and PNA. Counterstaining with Hoechst was applied. The rectangular area in the left upper image is enlarged in the right upper. GFP (green) and PNA (red) channels are independently shown in the lower images. (D) Haploid cells were observed in the dissociated sample of cultured testis tissues from the VAD-treated *Gsg2*-GFP mouse, 13 weeks old, on culture day 73. Dissociated cells were stained with antibodies against GFP (green) and SP56 (red), a sperm-specific protein that localizes to the sperm surface and in the acrosomal matrix. Nuclei were stained with Hoechst. (E) A sperm was found in a dissociated sample of cultured tissue from a VAD-treated *Acr*-GFP mouse, 23 weeks old, on culture day 50. It was stained with anti-GFP antibody along with nuclear staining by Hoechst. Scale bars, 1 mm (B); 50 μm (A, C); 10 μm (D, E).

In order to confirm the above-mentioned results, we prepared vitamin A-deficient (VAD) mice whose testes have undifferentiated spermatogonia alone as germ cells due to the arrest of spermatogonial differentiation on A_al_–A_1_ transition [16, 17]. Testes of VAD *Acr*- or *Gsg2-*GFP mice at birth and until around 5 weeks old showed GFP expression which corresponds with its histological feature of sound spermatogenesis, probably due to the vitamin A stored in the body. Then, the GFP expression gradually diminished and disappeared completely by 13 weeks old, which also corresponds with the cessation of spermatogenesis, leading to the almost total disappearance of differentiating germ cells (Fig 2A). We used testes of VAD mice at 13–21 weeks old which showed no GFP expression on explantation. Among 8 experiments, GFP expression was observed in all, and in 61 tissue pieces out of 72 in total (Fig 2B, Table 2). The cultured tissues contained round and elongating spermatids with GFP expression (Fig 2C). When cultured tissues were dissociated mechanically, we found isolated haploid cells (Fig 2D) and sperm in a single case (Fig 2E). These results again showed that our organ culture method is applicable for the induction of spermatogenesis from undifferentiated spermatogonia up to haploid cells in mature testis tissues of adult mice.

**Table 2:** Summary of VAD experiment.

Exp. ID	Age of mouse (weeks old)	GFP promoter	Medium supplement	Culture duration (weeks)	# Tissue Fragment cultured	# GFP(+) tissue	# GFP(-) tissue
1	14	Gsg2	KSR	10	14	9	5
2	14	Gsg2	KSR	7	8	7	1
3	17	Gsg2	KSR	11	7	7	0
4	17	Gsg2	AlbMAX	21	9	9	0
5	21	Gsg2	KSR	9	6	5	1
6	21	Gsg2	AlbMAX	9	9	8	1
7	14	Acr	KSR	9	10	7	3
8	14	Acr	KSR	11	9	9	0
					72	61	11

### Extent of spermatogenesis in adult and pup testis tissues

**Fig 3:**
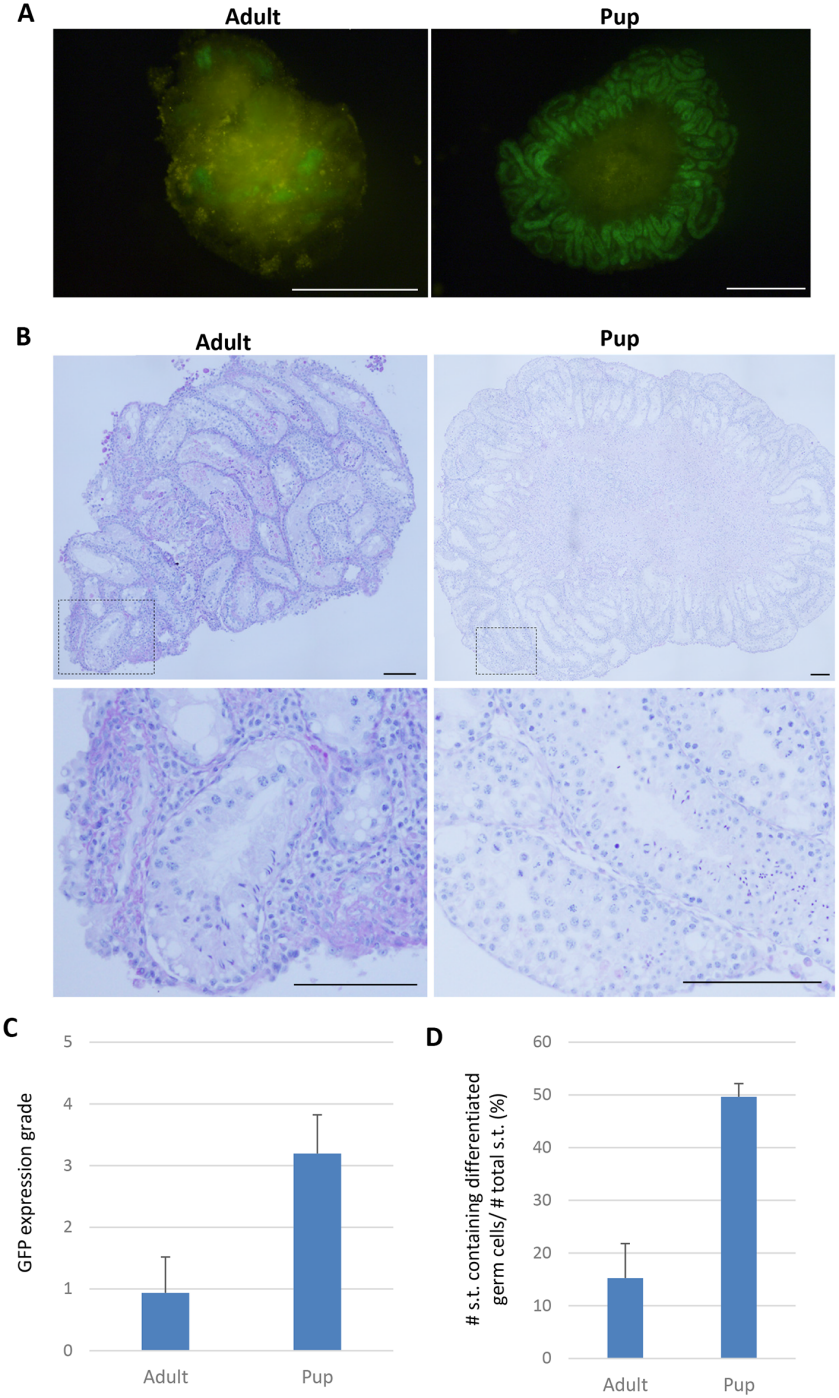
Extent of GFP expression in adult and pup testis tissues. (A) GFP expression images of testis tissues, from 2.5 dppp and seven-week-old Acr-GFP mice, cultured for 5 weeks. (B) PAS staining images of testis tissues, from 2.5 dppp and seven-week-old *Acr-*GFP mice, cultured for 5 weeks. The dotted rectangular areas are enlarged on the right. (C) Adults and pups were compared on the basis of the extent of *Acr*-GFP expression scored (judged at culture week 5). Testis tissue fragments of *Acr*-GFP mice, 2.5–4.5 dpp and 7–8 weeks old, were cultured for five weeks. Adult and pup testis tissues were examined in a total of 34 and 39 tissue pieces in three culture experiments, respectively (means±s.d., P<0.025 (Student’s t-test)). (D) The frequency of seminiferous tubules containing differentiated germ cells. Adult and pup testis tissues were examined in a total of 32 and 36 tissue pieces in three culture experiments, respectively. Sections stained with periodic acid Schiff were examined and the number of seminiferous tubules containing differentiated germ cells (spermatocytes, round spermatids, and/or elongating spermatids) asa well as seminiferous tubules not containing them were counted in each tissue to calculate the percentage of spermatogenesis-positive seminiferous tubule (means±s.d., P<0.002 (Student’s t-test)). Scale bars, 1 mm (A); 100 μm (B).

Although the culturing of adult mouse testis tissues demonstrated the progression of spermatogenesis, it’s extent appeared quite lower than that in the pup testis tissues cultured. To clarify the magnitude of their differences, we recorded the GFP expression extent, GFP grade, and performed thorough histological examination. Thirty-four adult testis tissues in 3 experiments and 39 pup testis tissues in 3 experiments were examined. The average GFP grade in adult and pup groups in the 5th culture week was 0.94 and 3.19, respectively, showing a significant difference (Fig 3A and 3C). The percentage of seminiferous tubules containing differentiated germ cells in these tissues was 15.2% in adult and 49.6% in pup groups (Fig 3B and 3D). Thus, the efficiency of spermatogenesis in adult testis tissues cultured was significantly lower than that of pup testis tissues.

### *Sl/Sl* ^*d*^ mutant adult mouse experiments

**Fig 4:**
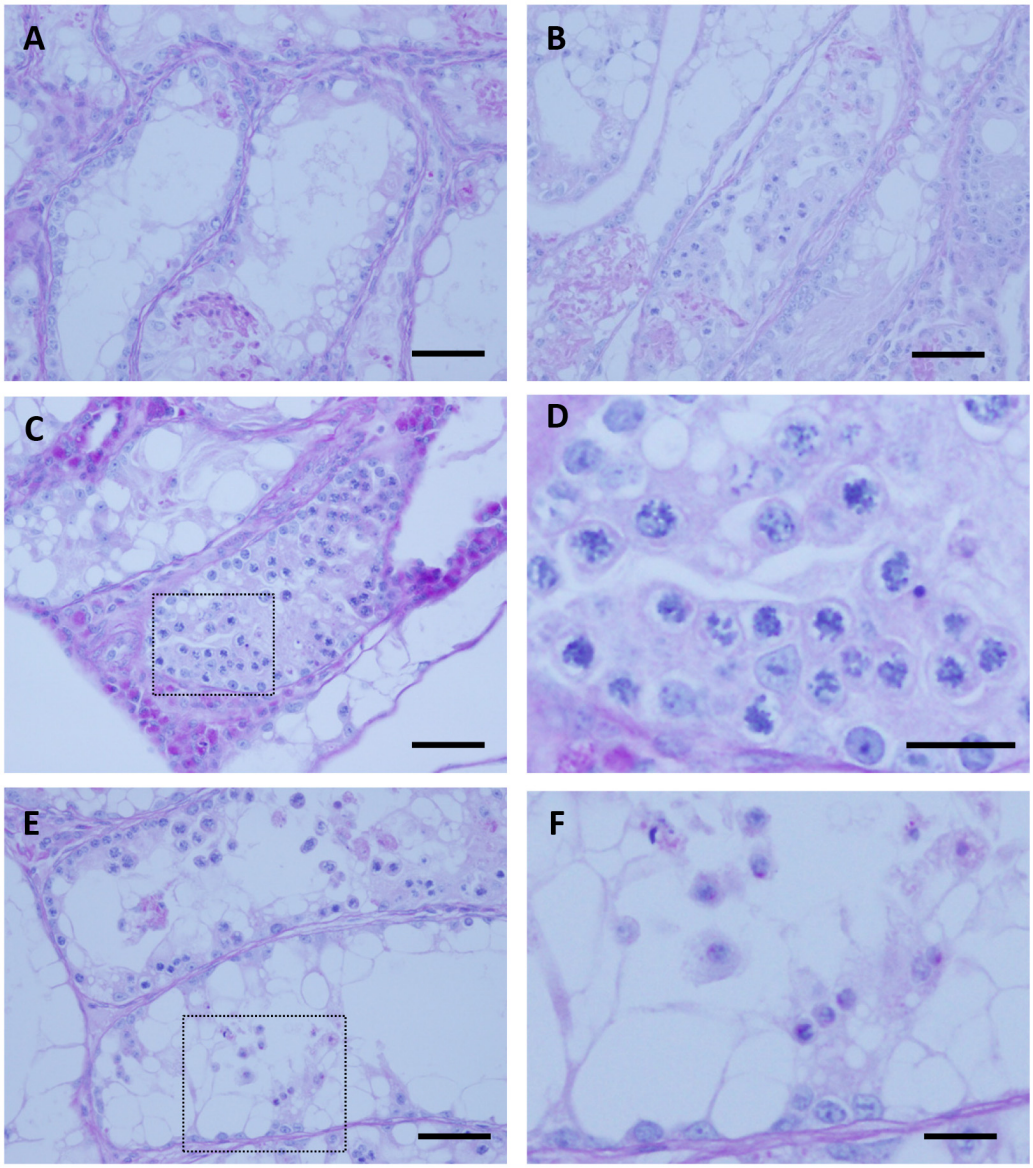
*Sl/Sl* ^*d*^ mutant adult mouse experiments. (A) Six-week-old *Sl/Sl* ^*d*^ mouse testis tissues, cultured for 45 days without KitL nor CSF-1 (control) showed no spermatogenesis. (B) The testis tissue of the same mouse showed differentiated germ cell accumulation in the seminiferous tubules when cultured with KitL (100 ng/mL). (C, D) When cultured for 40 days with KitL (100 ng/mL) plus CSF-1 (20 ng/mL), meiotic cells in the seminiferous tubules were observed. The rectanglar area in C is enlarged in D. (E, F) Culturing with KitL (500 ng/mL) for 38 days induced spermatogenesis up to round spermatids. The rectanglar area in E is shown in F, demonstrating several round spermatids with PAS-stained acrosomal caps or dots. Scale bars, 50 μm (A-C, E); 20 μm (D, F).

Lastly, we tested if spermatogenesis of *Sl/Sl* ^*d*^ mutant adult mice whose testes have only primitive spermatogonia as germ cells can be induced under our organ culture conditions. In our previous study, we showed that pup testis tissues with spermatogenic failure could be successfully treated *in vitro* by adding growth factors, KitL alone or in combination with colony stimulating factor-1 (CSF-1), to the culture medium [6]. The present study consisted of 7 experiments using one mouse on each occasion, culturing 69 tissue fragments in total (Table 3). We applied the same culture conditions used for pup mouse tissues. When they were cultured in medium without KitL, no progression of spermatogenesis was observed (Fig 4A). On the contrary, with KitL of 100 ng/mL or KitL 100 ng/mL plus CSF-1 20 ng/mL, seminiferous tubules containing meiotic cells appeared (Fig 4B–4D, Table 4). We increased the concentration of KitL up to 500 ng/mL and found a similar progression of spermatogenesis but no improvement was observed (Fig 4E, Table 4). Nonetheless, although rare, we found round spermatids with an acrosome dot stained red with PAS stain (Fig 4F). Taken together, these data suggest that the organ culture method can induce spermatogenesis in adult testis tissues just as in neonatal and pup mice, although the efficiency was lower and appearance of sperm was quite rare.

**Table 3:** Summary of adult *Sl/Sl* ^*d*^ mouse experiment, chronologically arranged.

Exp. ID	Medium supplement	Culture duration (days)	# Tissue fragment cultured	# Tissue with meiosis	# Tissue with round spermatids
1	Control	39	6	0	0
	KitL 100		11	6	0
2	Control	42	3	0	0
	KitL 100 + CSF1		7	0	0
3	Control	40	2	0	0
	KitL 100 + CSF1		5	1	1
4	Control	45	1	0	0
	KitL 100 + CSF1		1	0	0
5	Control	45	2	0	0
	KitL 100		4	1	1
	KitL 100 + CSF1		3	1	0
	KitL500 + CSF1		3	2	0
6	KitL500	38	3	1	1
	KitL 100 + CSF1		3	2	0
	KitL500 + CSF1		3	1	0
7	KitL 100 + CSF1	52	12	5	0
			69	20	3

**Table 4:** Summary of adult *Sl/Sl* ^*d*^ mouse experiment.

Medium supplements	# Experiment	# Tissue fragment cultured	# Tissue with meiosis	# Tissue with round spermatids
Control	5	14	0	0
KitL 100	2	15	7	1
KitL 100 + CSF1 20	6	31	9	1
KitL 500	1	3	1	1
KitL 500 + CSF1 20	2	6	3	0

## Discussion

In the present study, we have shown that our organ culture method is applicable for not only immature mouse testis tissues, but also mature adults for the induction of complete spermatogenesis. However, its efficiency was markedly lower in adult testis tissues. It has been reported that adult tissues, regardless of the organ source, are generally vulnerable to *ex vivo* conditions and difficult to maintain under those conditions [7–10]. Even though there have been several reports of the long-term culture of adult tissues, for weeks or months, the cultured tissues mostly exhibited significant changes in their histological architecture and cell composition [18, 19]. Accordingly, their functional properties may be lost, although there has been no report on this. Based on our present results, mainly focusing on the functional aspects of testis tissue, it was shown that adult tissues are vulnerable, compared to immature tissues, to the *ex vivo* conditions. Although mechanism is not clear, one plausible reason is that immature tissues are relatively resistant to hypoxic conditions, while adult tissues are not [20]. Thus, some studies used higher oxygen concentrations for culturing adult tissues. However, simply raising the oxygen content in the culture incubator reportedly did not overcome this problem [9]. In fact, there is always a trade-off between the demand for oxygen and its toxicity to cells and tissues [20]. Testis tissue is no exception, and is certainly sensitive to hypoxic conditions. It was reported that germ cells, from spermatogonia to spermatids, are far more vulnerable than somatic cells to hypoxic conditions and prone to apoptosis [21, 22]. It is noteworthy that adult testis tissues grafted in subcutaneous spaces of host animals showed degenerative changes and poor or no spermatogenesis, while immature testis tissues grew and matured to yield even complete spermatogenesis [23, 24]. For grafted tissues, vascularization is critical for their survival. The greater the extent and faster the development of vascularization, the more successfully graft tissues survive and show their original function at the grafted site. However, a previous study indicated a similar extent of vascularization both in adult and immature testis tissues [25]. Although not conclusive, it seems that higher susceptibility to a low oxygen concentration in adult tissues may be the primary reason to explain the difference in grafting results between adult and immature testis tissues [26]. In the present study, being able to omit the effect of vascularization, the results appeared to be very similar to those of grafting experiments. Thus, it emphasized the nature of adult tissue, which is vulnerable to ectopic conditions following grafting or culturing.

Besides a higher sensitivity to ischemia, adult tissues may have additional disadvantages reducing their *ex vivo* and *ex site* survival. It seems reasonable that the immature testis exhibits a type of flexibility to adapt to different conditions. For instance, an immature seminiferous tubule can grow from its original size to a size that a particular microenvironment will allow. On the other hand, adult seminiferous tubules have grown to a maximum size to produce the maximum number of sperm for a prolonged period. To transport those sperm out of the testis, the seminiferous tubules have fluid flowing inside which is produced by Sertoli cells and propelled by the coordinated cyclic contraction of the peritubular myoid cells [27, 28]. Such a highly sophisticated tissue architecture is sensitive to environmental changes and cannot maintain itself under such different conditions, a culture condition in this case. In order to effectively maintain adult testis tissue *in vitro*, therefore, a novel culture method is needed which can provide microenvironmental conditions much closer to those *in vivo*.

In the future, the organ culture technique for testis tissue could be applicable to many species, including humans. Then, it would be useful in clinical settings for the evaluation and treatment of male infertility. On such occasions, adult tissues should be dealt with more routinely than those of children. In the present study, we demonstrated that mouse spermatogenesis proceeds in adult tissue fragments. At the same time, however, its efficiency is not comparable to that in immature mice. Our results, therefore, could be a basis for future studies.
